# Isolation of dengue virus from the upper respiratory tract of four patients with dengue fever

**DOI:** 10.1371/journal.pntd.0005520

**Published:** 2017-04-05

**Authors:** Nai-Ming Cheng, Cheng Len Sy, Bao-Chen Chen, Tsi-Shu Huang, Susan Shin-Jung Lee, Yao-Shen Chen

**Affiliations:** 1 Division of Infectious Diseases, Department of Internal Medicine, Kaohsiung Veterans General Hospital, Kaohsiung, Taiwan; 2 Division of Microbiology, Department of Pathology and Laboratory Medicine, Kaohsiung Veterans General Hospital, Kaohsiung, Taiwan; 3 Faculty of Medicine, National Yang-Ming University, Taipei, Taiwan; University of California, Berkeley, UNITED STATES

## Abstract

**Background:**

Dengue fever is an important arboviral disease. The clinical manifestations vary from a mild non-specific febrile syndrome to severe life-threatening illness. The virus can usually be detected in the blood during the early stages of the disease. Dengue virus has also been found in isolated cases in the cerebrospinal fluid, urine, nasopharyngeal sections and saliva. In this report, we describe the isolation of dengue virus from the upper respiratory tract of four confirmed cases of dengue.

**Methods:**

We reviewed all laboratory reports of the isolation of dengue virus from respiratory specimens at the clinical microbiology laboratory of the Kaohsiung Veterans General Hospital during 2007 to 2015. We then examined the medical records of the cases from whom the virus was isolated to determine their demographic characteristics, family contacts, clinical signs and symptoms, course of illness and laboratory findings.

**Results:**

Dengue virus was identified in four patients from a nasopharyngeal or throat culture. Two were classified as group A dengue (dengue without warning signs), one as group B (dengue with warning signs) and one as group C (severe dengue). All had respiratory symptoms. Half had family members with similar respiratory symptoms during the period of their illnesses. All of the patients recovered uneventfully.

**Conclusions:**

The isolation of dengue virus from respiratory specimens of patients with cough, rhinorrhea and nasal congestion, although rare, raises the possibility that the virus is capable of transmission by the aerosol route among close contacts. This concept is supported by studies that show that the virus can replicate in cultures of respiratory epithelium and can be transmitted through mucocutaneous exposure to blood from infected patients. However, current evidence is insufficient to prove the hypothesis of transmission through the respiratory route. Further studies will be needed to determine the frequency of respiratory colonization, viable virus titers in respiratory secretions and molecular genetic evidence of transmission among close contacts.

## Author summary

Dengue virus is rarely identified in respiratory specimens. We retrospectively identified four patients with dengue fever who had the virus isolated from their nose or throat. All the patients had respiratory signs or symptoms. Half had family members who also had respiratory symptoms. Further studies are needed to evaluate the possibility of respiratory transmission of this virus.

## Introduction

Dengue fever is a mosquito-borne disease caused by dengue virus (DENV). There are four serotypes–DENV-1, DENV-2, DENV-3 and DENV-4. Dengue is widely distributed throughout tropical and sub-tropical areas around the world and imposes great economic burden. It is transmitted by *Aedes aegypti* and less commonly by *Aedes albopictus*. The World Health Organization (WHO) estimates that up to 100 million infections occur annually [1]. The incidence of dengue fever has increased 30-fold over the past 50 years, making it one of the most important arboviral disease around the world [1].

The course of dengue usually go through three phases: febrile phase, critical phase and recovery (convalescence) phase [1, 2]. Febrile phase represents the beginning of high fever caused by dengue viremia, which lasts for 2–7 days. Critical phase is featured by signs of plasma leak, such as pleural effusion, ascites, petechiae or intracranial bleeding around the time of defervescence, which lasts for 24–48 hours. After patients survive the critical phase, they go through convalescent phase and recover spontaneously.

Timely and efficient diagnosis is crucial to the management of dengue. Current diagnostic tests enable simple, rapid and sensitive detection of the virus. This is achieved by isolation of the virus or serologic or molecular methods depending on the phase of the disease [3]. Nonstructural 1 (NS1) protein is a glycoprotein encoded by dengue virus and secreted from dengue-infected cells [4]. Detection of the NS1 antigen by enzyme-linked immunosorbent assay in the acute phase is an important tool for prompt diagnosis of dengue [5]. Confirmation is made by reverse-transcriptase polymerase chain reaction (RT-PCR) from viremic specimens [1]. Rapid diagnostic tests for dengue antibodies (IgG, IgM) are also commercially available.

Although the early diagnosis of dengue fever is usually based on detection of viremia, there are several reports of DENV in cerebrospinal fluid [6], saliva and urine [7]. We are aware of only one case report of the isolation of dengue virus from a respiratory specimen [8]. In this report, we describe four cases of dengue fever in which the virus was isolated from the upper respiratory tract.

## Methods

### Ethics statement

This study was approved by the institutional review board of the Kaohsiung Veterans General Hospital. Patient data were anonymized.

### Study design

The study was conducted at the Kaohsiung Veterans General Hospital, in southern Taiwan. We identified all isolations of dengue virus from respiratory specimens obtained from patients admitted to the emergency service, hospital or clinics setting during 2007 to 2015. A respiratory specimen was defined as material obtained from the sputum, nasopharyngeal or pharyngeal secretions, or bronchoalveolar lavage fluid. The medical records of each patient were reviewed to provide demographic data, clinical signs and symptoms, contact history, laboratory findings, clinical course and outcome.

### Virus isolation and growth

Respiratory specimens were sent to the virology laboratory for identification. They were inoculated into six different cell lines: human lung fibroblast (MRC-5), human rhabdomyosarcoma (RD), human lung adenocarcinoma (A549), African green monkey kidney cell (Vero), Rhesus monkey kidney (MK2), and Madin Darby canine kidney (MDCK). Fresh Eagle minimal essential medium was added and incubated at 35°C with 5% CO_2_. Observations were made twice a week for 3 weeks at 4x magnification until a cytopathic effect (CPE) was evident.

### Indirect immunofluorescence assay

CPE positive cultures were identified using indirect immunofluorescence assay (IFA) screening kits for respiratory viruses (Catalogue number 3360, Chemicon International Inc.) and enteroviruses (Catalogue number 5007, Chemicon International Inc.) according to the manufacturer’s instructions. Respiratory IFA contains antibodies which recognize adenovirus, influenza A and B, parainfluenza 1, 2 and 3, and respiratory syncytial virus. Enterovirus IFA contains antibodies which recognize echoviruses, coxsackie A and B, polio 1,2, and 3, and enteroviruses 70 and 71.

### Reverse transcription and PCR amplification

Viral RNA was extracted from each culture supernatant using the QiAmp viral RNA kit (Qiagen inc.) according to the manufacturer’s instructions and analyzed using classic RT-PCR. We used primer DV1 and type-specific DSP1, DSP2, DSP3 and DSP4 according to a previous study [9]. One-step RT-PCR assays (SuperScript^TM^ One-Step RT-PCR with Platinum Taq kit; Invitrogen Corporation) were performed. The amplification reaction consisted of cDNA synthesis at 50°C for 30 minutes; predenaturation at 95°C for 1 minute, PCR amplification for 45 cycles at 95°C for 30 seconds, at 50°C for 30 seconds, and at 72°C for 7 minutes; followed by a final extension step at 72°C for 7 minutes. PCR products were analyzed by electrophoresis on a 1.5% agarose gel; the expected amplicons were 169 base pair (bp) for DENV-1, 362 bp for DENV-2, 265 bp for DENV-3, and 426 bp for DENV-4.

### Statistics

Descriptive statistics were used to characterize the study population, clinical features and laboratory findings.

## Results

Our virology laboratory received 31893 respiratory specimens during 2007–2015. Dengue virus was isolated from either the nasopharynx or the throat of 4 patients. Their medical records were reviewed and summarized below.

### Patient 1

A 64-year-old otherwise healthy woman presented to the emergency department with fever of 38.0°C for one day in November 2015. This was associated with generalized myalgia, severe headache, rhinorrhea, nasal congestion, productive cough and gingival bleeding. She had no signs of respiratory distress when seen in the emergency department. Laboratory analysis revealed white blood cell count (WBC) of 3.18×10^9^/L, hematocrit (HCT) of 35.9% and a platelet count of 166×10^9^/L. She tested positive for serum dengue NS1 antigen and negative for dengue IgM and IgG (SD BIOLINE Dengue Duo). A quick test (BD Veritor^TM^ System For Rapid Detection of Flu A+B) was positive for influenza A. She was treated with oral oseltamivir and subsequently hospitalized. A nasopharyngeal culture obtained after admission was positive for DENV-2. Her condition gradually improved and she was discharged home after 5 days of hospitalization.

### Patient 2

A 17-year-old young man presented to the pediatric emergency department with intermittent high-grade fever for two days in August 2015. This was associated with myalgia, headache, rhinorrhea, sore throat, productive cough and abdominal pain. Physical examination showed a congested oropharynx and soft abdomen with hyperactive bowel sounds. Laboratory tests showed a WBC of 3.06×10^9^/L, a HCT of 38.6% and a platelet count of 151×10^9^/L. Throat swab tested negative for influenza quick test and negative for beta-hemolytic streptococcus and nasopharyngeal swab was positive for DENV-2. His stool was negative for adenovirus (Adenolex test, Orion Diagnostica, Finland, as text in S1). He was treated for acute bacterial tonsillitis with oral amoxicillin and followed up in the pediatric clinic 2 days after, with marked improvement of symptoms.

### Patient 3

A 14-year-old girl presented to the emergency department with fever, chills and fatigue for 3 days in September 2010. She had history of pneumonia and acute otitis media (AOM) when she was in elementary school. Her aunt, living with her, had similar signs and symptoms. She had no recollection of a mosquito bite. Her temperature was 38.9°C. Her conjunctivae and throat were congested. Laboratory investigations showed a WBC of 2.78×10^9^/L, a platelet count of 139×10^9^/L and HCT of 36.2%. A nasopharyngeal influenza quick test was negative. She was discharged with antipyretics, but returned the next day due to nausea, persistent fever and new-onset bilateral otalgia. Her eardrums were normal without discharge. There was no skin rash. She was treated with amoxicillin-clavulanate and antipyretics. A serologic test at day 4 was positive for dengue NS1 antigen. The throat swab collected at day 5 of fever showed a cytopathic effect. The virus culture was positive for DENV-2 by RT-PCR. She became afebrile on day 6 and gradually improved.

### Patient 4

A 12-year-old boy presented to the pediatric emergency room with a temperature of 40°C for one day in October 2008. This was associated with generalized weakness, myalgia, severe headache and vomiting. His father had similar symptoms and was admitted in our hospital at the same time. The child’s blood pressure was 178/78 mmHg and heart rate was 133/min. He had a congested pharynx with bilateral coarse breath sounds. Laboratory test showed a WBC of 8.49×10^9^/L, HCT of 41.5% and platelet count of 264×10^9^/L. Chest X-ray was unremarkable. He was admitted to the pediatric service and treated with oseltamivir. His fever and other signs and symptoms persisted for 4 days and were accompanied by lethargy, severe bone pain, generalized skin rashes and one episode of epistaxis. Laboratory data revealed a WBC of 2.08×10^9^/L, a platelet count of 70×10^9^/L and hemoconcentration with HCT of 46.9%. The patient was transferred to the pediatric intensive care unit for monitoring because of signs of severe dengue. A nasopharyngeal influenza quick test was negative. Throat swab and blood virus cultures were obtained at day 4 of illness. DENV-1 was isolated from both specimens. Abdominal sonography and chest-x ray showed no signs of pleural effusion or ascites. The patient was treated supportively and was discharged at day 12 of illness.

### Summary

Their clinical findings are summarized in Table 1 and virology data in Tables 2 and 3.

**Table 1:** Clinical and serologic findings in four patients from whom dengue virus was isolated from their throat or nasopharynx.

Patient	1	2	3	4
Age	64	17	14	12
Sex	F	M	F	M
Clinical Symptoms	Fever Myalgia Headache	Fever Myalgia Abdominal pain	Fever Nausea	Fever Myalgia Headache Vomiting Bone pain
Respiratory Symptoms	Cough Rhinorrhea Nasal congestion	Cough Rhinorrhea Sore throat	Cough Rhinorrhea	Rhinorrhea Nasal congestion
Clinical Diagnosis	Dengue	Acute tonsillitis	Suspected otitis media or dengue	Dengue
Dengue Serology	NS1 (+), IgM (-), IgG (-)	Not tested	NS1 (+), IgM (-), IgG (-)	NS1 (+), IgM (-), IgG (-)
Dengue Warning Signs	Gingival bleeding	Nil	Nil	Gingival bleeding Epistaxis Petechiae Purpura
Clinical Specimen Cultured	Nasopharyngeal swab	Throat swab	Throat swab	Throat swab
^a^Day of Illness during virus isolation	Day 2, febrile phase	Day 1, febrile phase	Day 4, febrile phase	Day 4, critical phase
Dengeue virus Serotype	Dengue virus type 2	Dengue virus type 2	Dengue virus type 2	Dengue virus type 1
^b^Case Classification	Dengue with warning signs	Dengue without warning signs	Dengue without warning signs	Severe Dengue

^a^Definition of dengue phase was based on 2009 WHO Dengue Guideline.

^b^Classification was based on the 2009 WHO Dengue Guideline.

**Table 2:** Description of cell lines with cytopathic effects to dengue virus from respiratory specimens of four patients with dengue.

Patient number	Clinical specimens	Cells with ^a^CPE	Day till CPE observed	^b^CPE Level	^c^Cell Line Passage
1	Nasopharyngeal swab	Vero	8 days	2+	0
		MK2	11 days	1+	0
2	Throat swab	Vero	12 days	3+	0
3	Throat swab	Vero	11 days	1+	0
		MK2	24 days	1+	0
4	Throat swab	MK2	21 days	1+	0

^a^CPE: cytopathic effect

^b^CPE level: 1+/2+/3+/4+ indicating 25%/50%/75%/100% of cells showed CPE.

^C^0: indicating no subculture required for positive CPE.

**Table 3:** Results of Immunofluorescence assay and RT-PCR of specimen from positive-CPE inoculated cells in 4 patients with dengue virus culture-positive respiratory specimens.

Patient number	CPE-positive cell	Test	Test Result
1	Vero cell	Respiratory virus ^a^IFA	Negative
		Enterovirus IFA	Negative
		Dengue RT-PCR	Dengue virus type 2
2	Vero cell	Enterovirus IFA	Negative
		Dengue RT-PCR	Dengue virus type 2
3	MK2 cell	Respiratory virus IFA	Negative
		Enterovirus IFA	Negative
		Dengue RT-PCR	Dengue virus type 2
4	MK2 cell	Respiratory virus IFA	Negative
		Enterovirus IFA	Negative
		Dengue RT-PCR	Dengue virus type 1

^a^IFA: Immunofluorescence assay

## Discussion

Four cases of confirmed dengue fever with isolation of dengue virus from their respiratory tract was presented. Their clinical manifestation ranges from mild illness, which can be managed at outpatient clinics, to severe dengue, which required closely monitoring in ICU setting. All patients had respiratory tract symptoms and half of their family members also reported respiratory symptoms. This suggests the possibility of the respiratory route for transmission of dengue virus. We reviewed the literature for evidence supporting this concept.

### Current understanding of dengue transmission

Dengue virus is typically transmitted by means of its vector mosquitos. Humans are its primary amplifying host [1]. The incubation period after being bitten by a mosquito falls between 3–10 days in 95% of the patients [10]. After this period, DENV begins to replicate rapidly, causing viremia for an average of 5.1 days for primary infection and 4.4 days for secondary infection [11]. A mosquito can acquire DENV through ingestion of viremic blood that has high circulating virus [12]. Once the virus is acquired, the mosquito remains infected throughout its life and can pass it to its offspring through transovarial transmission [13]. Transovarial transmission of DENV 1–4 has been observed in both *A*. *aegypti* and *A*. *albopictus* [14], making vector population control a crucial issue in the management of dengue outbreak.

Dengue virus can also be transmitted from infected blood by transfusions [15], organ transplantation [16–18], vertical transmission from mother to fetus [19] and needle stick injury [20].

### Aerosol transmission

There has only been one prior report, to our knowledge, of the isolation of DENV from nasal and pharyngeal swabs of a patient residing in a dengue-endemic area [8]. We now add four more similar cases. Dengue fever is commonly associated with respiratory symptoms. A dengue surveillance system found that 35–38% of laboratory-positive dengue patients had sore throat, cough and nasal congestion [21]. This raises the possibility that dengue can be transmitted via respiratory aerosols or by direct contact from patients with respiratory infection. Half of the patients had family members who also had similar signs and symptoms during onset of their illness. The finding of dengue virus in their nasopharynx and throats and the close association of two of them with symptomatic relatives made us wonder whether respiratory aerosols might have transmitted dengue virus into the air or by close contact. Possible aerosol transmission has been reported with other flaviruses, including Zika virus [22], tick-borne encephalitis virus [23] and Wesselsbron virus [8].

There is strong evidence from work conducted on dengue fever in World War II that dengue virus can be transmitted through nasopharyngeal exposure to infected serum [24]. Seventy-five percent of patients receiving intranasal instillation of infected serum developed febrile illness 11 days after inoculation. The median onset of leukopenia was slower compared to patients infected by mosquito-bites (10.5 days vs. 8 days). The duration of fever and leukopenia were also shorter in patients infected intranasally. Half of the intranasal-infected patients developed epistaxis compared to 6% of patients infected by mosquito bites or infected serum (P<0.001) [25].

There are other reports of possible mucocutaneous transmission of dengue virus. A health care worker was diagnosed with dengue after she accidentally splashed the infected serum on her face, including her eye, nose and mouth. She had nosebleeds, anorexia and eye pain 10 days afterwards. Serological markers indicated acute dengue infection [26]. Another investigator was infected with the virus while performing a laboratory experiment involving primary infection of colony mosquitoes with dengue virus via an artificial membrane feeding apparatus. Nucleotide sequencing found 99.8% homology between the virus retrieved from patient and the laboratory strain. The patient was wearing gloves, gowns and eye protection during the procedure. It was postulated that the virus may have been transmitted mucocutaneously via virus-infected aerosolized blood droplets or through an unrecognized dermal abrasion [27].

### Ability of dengue virus to grow in respiratory epithelial cells

Before the availability of polymerase chain reaction, typical viral isolation techniques for dengue virus involved inoculating specimens in C6/36 cells, larva tissue of *A*. *Albopictus* [28]. Our virology laboratory observed cytopathic effect in either Vero or MK2 cell lines. Aside from these cell lines, all four serotypes of DENV have been demonstrated to be able to infect and replicate in human primary lung epithelium and lung cancer cell lines [29]. These findings indicate that respiratory epithelium may be a possible target of dengue virus.

The cells routinely used for viral culture of respiratory specimens in our laboratory were designed to detect common respiratory pathogen rather than dengue virus. Once inoculated cells show CPE, we would use screening IFA for respiratory virus and enteroviruses. Coinfections with respiratory virus and enterovirus IFA were excluded since all our specimens tested negative for these pathogens by IFA.

### Limitation

Since this is a retrospective study, it has certain limitations. The respiratory secretions of dengue–infected patients were not routinely cultured for viral growth. A review of 281 cases of patients with dengue in our hospital in 2015 shows that only 2 patients had respiratory specimens sent for viral culture. The culture cells we used were not optimal for dengue virus growth and may underestimate dengue infection. Of 31893 specimens received, only 4 tested positive for dengue virus. Testing for dengue PCR from respiratory virus and enterovirus IFA-negative specimens were done only when dengue virus was suspected by the microbiologists according to their own experiences.

### Conclusion

This report of the isolation of dengue virus from the airway secretions of four patients with respiratory symptoms raises the possibility that it may spread by the aerosol route or by direct contact. This concept is supported by studies which show that the virus can replicate in cultures of respiratory epithelium and can be transmitted through mucocutaneous exposure to blood from infected patient. Current evidences are insufficient to prove the hypothesis of transmission through the respiratory route. Further studies are needed to determine the frequency of respiratory colonization in patients with dengue, viable virus titers in respiratory secretions and molecular genetic evidence of transmission of the same strain among close contacts.

## Supporting information

S1 Tests Details about tests performed in our patients. (DOCX)
