# Supplementary material for: Isolation of dengue virus from the upper respiratory tract of four patients with dengue fever
Source: PLoS Negl Trop Dis. 2017 Apr 5;11(4):e0005520. doi: 10.1371/journal.pntd.0005520 (PMC5403165; doi:10.1371/journal.pntd.0005520)
Supplement: S1 Tests — (DOCX) [file pntd.0005520.s001.docx]

S1 Tests

# Influenza Quick Test

1. **Methods**: Chromatographic immunoassay, qualitative detection.
2. **Manufacturer**: BD Veritor^TM^ System For Rapid Detection of Flu A+B.
3. **Performance**: Sensitivity and specificity was 80 and 94 % respectively.
4. **References**: BMC Infect Dis. 2016 Sep 9;16:481. doi: 10.1186/s12879-016-1811-9
5. **Instruction manual**: <http://www.bd.com/ds/technicalCenter/inserts/8087667(12).pdf#page=1&view=Fit>

# Dengue NS1/IgG/IgM Serology

1. **Methods**: in-vitro immunochromatographic, qualitative detection.
2. **Manufacturer**: SD BIOLINE Dengue Duo (Dengue NS1 Ag + IgG/IgM)
3. **Performance**: Sensitivity : 92.4% (Dengue NS1 Ag), 94.2% (Dengue IgG/IgM); Specificity : 98.4%(Dengue NS1 Ag), 96.4% (Dengue IgG/IgM)
4. **Instruction manual**: <file:///C:/Users/bigna/Downloads/LEF11EN%20v2%20Dengue%20Brochure%20EN.pdf>

# Adenovirus Quick test

1. **Methods**: latex agglutination test
2. **Manufacturer**: Adenolex test, Orion Diagnostica, Finland
3. **Performance**: Compared to ELISA and EM, the sensitivity was 100% and 95% respectively, and the specificity 100%
4. **References**: J Med Virol. 1987 Dec;23(4):311-6. PMID: 2826673
